# HRCT findings of pulmonary sarcoidosis; relation to pulmonary function tests

**DOI:** 10.1186/2049-6958-8-8

**Published:** 2013-02-05

**Authors:** Fatih Ors, Seyfettin Gumus, Mehmet Aydogan, Sebahattin Sari, Samet Verim, Omer Deniz

**Affiliations:** 1 Department of Radiology, Gulhane Military Medical Academy, Ankara, Etlik, 06010, Turkey; 2 Department of Pulmonary Medicine, Gulhane Military Medical Academy, Ankara, Etlik, 06010, Turkey; 3 Mevki Military Hospital, Radiology Service, Ankara, Diskapi, Turkey

## Abstract

**Background:**

Chest-X-ray has several limitations in detecting the extent of pulmonary disease in sarcoidosis. It might not reflect the degree of pulmonary involvement in patients with sarcoidosis when compared to computed tomography of the thorax. We aimed to investigate the HRCT findings of pulmonary sarcoidosis and to find out the existence of possible relations between HRCT findings and PFTs. In addition, we aimed to investigate the accordance between HRCT findings and conventional chest-X-ray staging of pulmonary sarcoidosis.

**Method:**

45 patients with sarcoidosis with a mean age 29.7+/− 8.4 years were evaluated. Six of them were female and 39 were male. The type, distribution and extent of the parameters on HRCT/CTs were evaluated and scored. Chest-X-rays were evaluated for the stage of pulmonary sarcoidosis. Correlations were investigated between HRCT/CT parameter scores, Chest X-Ray stages and pulmonary function parameters.

**Results:**

Nodule, micronodule, ground glass opacity and consolidation were the most common HRCT findings. There were significant correlations between pulmonary function parameters, HRCT pattern scores, and chest-X-ray stages. A significant correlation between chest-x-ray score and total HRCT score was found.

**Conclusions:**

Pulmonary sarcoidosis patients might have various pulmonary parenchymal changes on HRCT. Thorax HRCT was superior to chest-X-ray in detecting pulmonary parenchymal abnormalities. The degree of pulmonary involvement might be closely related to the loss of pulmonary function measured by PFTs. Chest-X-ray is considered to have a role in the evaluation of pulmonary sarcoidosis.

## Background

Sarcoidosis is a systemic granulomatous disease of unknown etiology mainly involving the mediastinum and the lungs. The diagnosis is usually based on typical radiological findings as bilateral symmetric hilar lymphadenopathies, demonstration of non-caseating granulomas along with the exclusion of other known granulomatous diseases [1-4]. More than one organ or system involvement at the time of diagnosis is a usual feature of the disease. Even though it is a chronic disorder, remissions during the course of the disease are common especially in the lower radiologic stages of the disease [1-5]. Mainly for this reason, in clinical practice the treatment with steroids is indicated for patients with severe pulmonary disease. In addition, steroid treatment might also change the natural course of the disease [1,4,6]. The borders of the decision making in pulmonary sarcoidosis are not certain, and in some patients with sarcoidosis making the decision of steroid treatment might be troublesome. This is usually the case for pulmonary sarcoidosis patients having borderline pulmonary function test (PFT) results since a treatment decision for pulmonary sarcoidosis mainly based on the degree of impairment in pulmonary function tests and/or the radiological extent of pulmonary disease [1,7]. Chest-X-ray has several limitations in detecting the extent of pulmonary disease in sarcoidosis. It might not reflect the degree of pulmonary involvement in patients with sarcoidosis when compared to computed tomography of the thorax [5,7-9]. PFTs are generally used to determine the degree of impairment in pulmonary function in many diseases. However, there are not enough data about whether PFTs reflect the degree of pulmonary parenchymal involvement in pulmonary sarcoidosis. Mainly for these reasons, we aimed to investigate the HRCT findings of pulmonary sarcoidosis and to find out the existence of possible relationships between HRCT findings and PFTs. In addition, we aimed to investigate the accordance between HRCT findings and conventional chest-X-ray staging of pulmonary sarcoidosis.

## Method

Medical records of the patients with pulmonary sarcoidosis between 2005 and 2012 were screened retrospectively. Patients having a diagnosis of sarcoidosis were included into the study. Except for 4 of them, all the PFTs and CT scans (CTs) were performed within one month. For 4 patients the aforementioned procedures were performed within 2 months. Patients having no retrieved PFTs or thorax CTs were excluded from the study. As a result, 45 patients with sarcoidosis, with a mean age 29.7 ± 8.4 years, were evaluated. There were 6 females (mean age:41.8 ± 12.6) and 39 males (mean age:27.9 ± 5.9).

**Figure 1:**
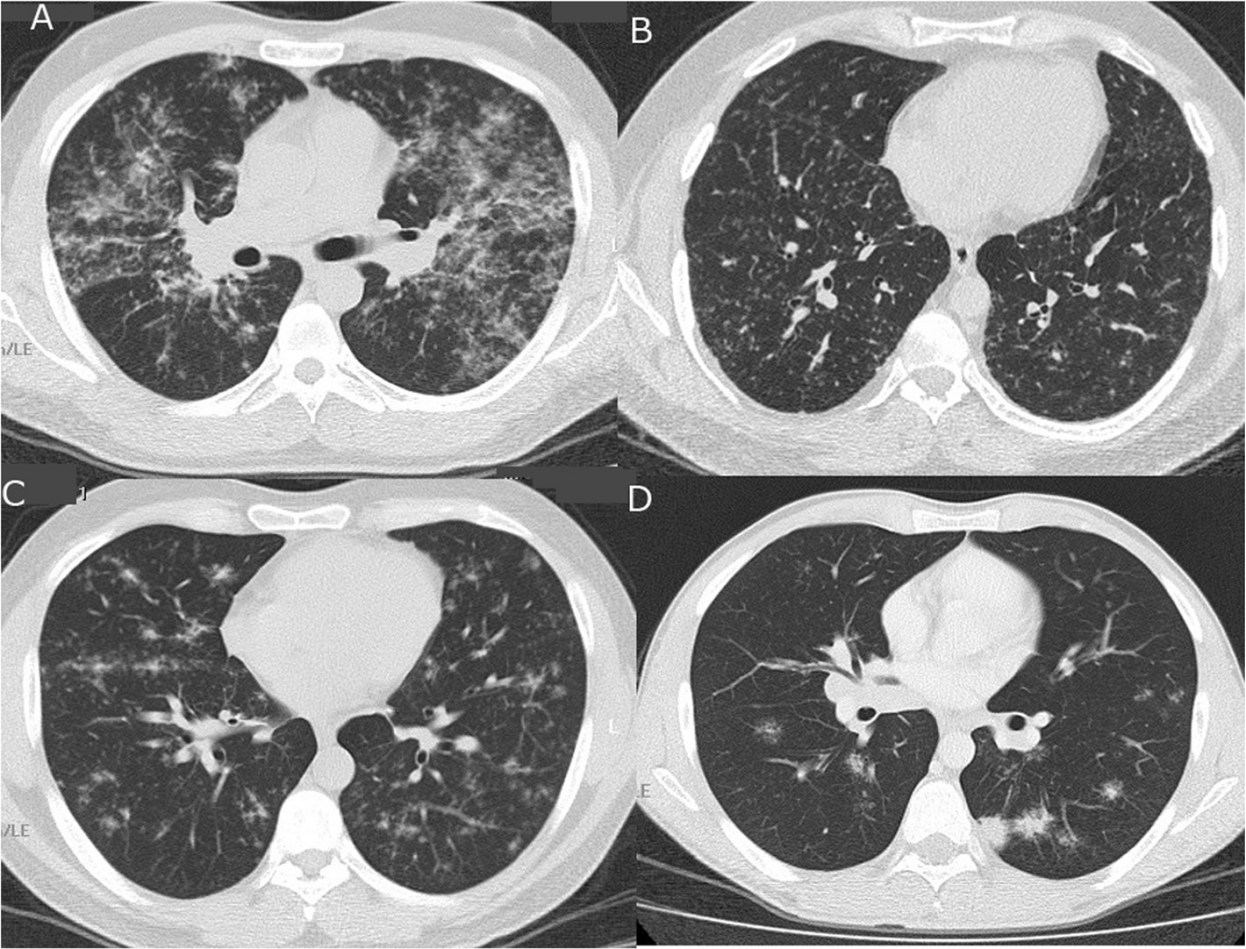
Some HRCT patterns (A: Ground glass opacity, B: Micronodule, C: Nodule, D: Consolidation).

The scoring and the evaluation of CTs and chest X rays were performed by two radiologists. If there was any disagreement between them, their final consensus score was recorded. HRCTs of 40 patients and thorax CTs of 5 patients were evaluated. CTs were performed at 5 mm section interval and HRCTs were performed 10 mm section interval (120 kV, 175 mA), [1 mm slice thickness, 1.5 s scanning time] using the GE Medical System HiSpeed CT/i (Milwaukee, USA) or the 16-slice MDCT (Philips, MX 8000 IDT 16, Best, The Netherlands) machines. The following and previously defined [10] HRCT patterns were evaluated: nodule (N), micronodule (MN), consolidation (C), ground glass opacity (GGO) (Figure 1), parenchymal band (PB), centrilobular emphysema, panacinar emphysema, paraseptal emphysema, bronchiectasis, interlobular septal thickening (ILST), intralobular septal thickening (intraLST) and subpleural interstitial thickening (SPIT). The distribution and extent of the parameters on HRCTs or on thorax CTs were determined as previously defined [11] and as following: parenchymal areas above the main carina were upper zones, areas below lower pulmonary veins were lower zones and areas between two zones were the middle zones of each lung. The degree of involvement was scored as following : no involvement 0 point, involvement up to 25% of a previously defined lung zone 1 point, involvement between 25% and 50% of a lung zone 2 points, involvement between 50% and 75% of a lung zone 3 points and involvement more than 75% of a lung zone 4 points. Thus for every single parameter, a patient might have 24 points. The sum of the scores of every single parameter for each patient was defined as total HRCT score.

We could retrieve chest-X-rays of 42 patients. Chest-X-rays of the patients were also evaluated for the stage of pulmonary sarcoidosis [1,4,7]. Twenty two patients were classified as radiologic stage 1, 14 patients as stage 2, 4 patients as stage 3 and 2 patients as stage 4. Patients were compared for total HRCT scores according to their radiological stages by using One-way ANOVA test.

The localizations of lymph nodes were determined and documented as hilar, mediastinal, paratracheal, para-aortic and subaortic. In one patient the localization of lymph nodes was not determined because the mediastinum was not visualized due to type of the radiologic technique. The degree of lymph node involvement was also evaluated and scored as mild, moderate and severe for each patient [7].

Spirometric parameters and diffusion parameters were measured by using either Vmax encore system (VIASYS Healthcare) or Cosmed Quark PFT 1 system. In 29 patients both spirometric measurements and diffusion measurements were done by using Vmax encore system. In five patients spirometric measurements were done by using Quark PFT 1 and diffusion measurements were done by using Wmax encore system. In 11 patients only spirometric measurements were done by using Quark PFT 1 system. “ERS 93” was used for predicted values in Quark PFT 1 system. “ERS 1993 Update + Zapletal” was used for predicted values in Vmax encore system.

Correlations were investigated between the results of pulmonary function measurements and the score of every single HRCT/CT parameter, the total HRCT scores and the chest-X-ray scores. A correlation was investigated between total HRCT scores and chest-X-ray scores. Pearson correlation test for parametric variables and Spearman correlation test for non parametric variables were used.

## Results

**Figure 2:**
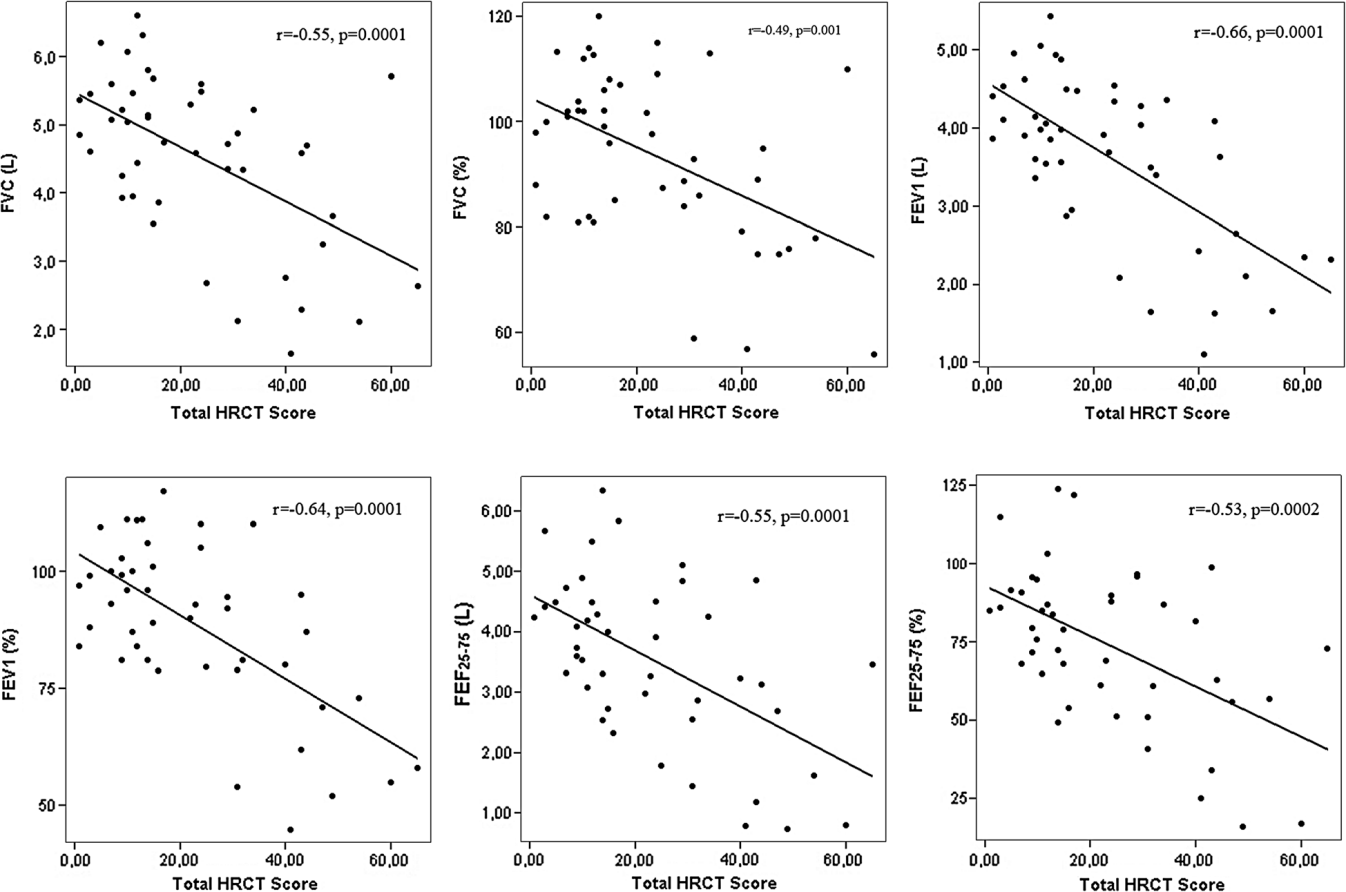
Correlations between total HRCT scores and spirometric values displayed with scatter plot graphics.

HRCT showed a wide spectrum of patterns: in some patients the pulmonary parenchyma was nearly clear, while in others almost all kinds of HRCT patterns were found. The amount of HRCT patterns are listed in Table 1. N, MN, GGO and consolidation were the most common HRCT findings. Spirometry and lung diffusion results are shown in Table 2. There were significant correlations between pulmonary function parameters and total HRCT scores, and HRCT pattern scores (Table 3). In Figure 2 correlations between total HRCT scores and spirometric values are displayed with scatter plot graphics.

**Table 1:** The amount of HRCT patterns

	**N**	**Minimum**	**Maximum**	**Mean**	**SD**
MN	41	1	21	7,3	4,5
N	37	1	10	4,5	2,3
C	21	1	10	4,1	2,8
GGO	36	1	12	4,8	2,8
BL	16	1	9	3,5	2,2
CLE	1	1	1	1	.
PANE	5	2	3	2,2	0,4
PSE	3	1	5	2,3	2,3
ILST	7	1	4	2,7	1,1
IntraLST	4	1	4	2	1,4
SPIT	23	1	2	1,8	0,3
PB	24	1	11	4,5	3,1
Cavity	1	1	1	1	.
BVB	27	1	4	1,9	0,6
Air cyst	1	1	1	1	.

*BL*, Bronchial lesions; *BVB*, Bronchovascular bundle; *C*, Consolidation; *CLE*, Centrilobular emphysema; *GGO*, Ground glass opacity; *ILST*, Interlobular septal thickening; *IntraLST*, Intralobularseptal thickening; *MN*, Micronodule; *N*, Nodule; *PANE*, Panacinar emphysema; *PB*, Parenchymal band; *PSE*, Paraseptal emphysema; *SPIT*, Subpleural interstitial thickening; *SD*, Standard Deviation.

**Table 2:** Spirometry and lung diffusion values of the patients

	**n**	**Mean (SD)**	**Minimum**	**Maximum**
**FVC (L)**	45	4.56 (1.22)	1.65	6.61
**FVC (%)**	45	93.9 (15.8)	56.0	120.0
**FEV**_**1**_**(L)**	45	3.63 (1.04)	1.11	5.43
**FEV**_**1**_**(%)**	45	88.6 (17.6)	45.0	117.0
**FEV**_**1**_**/FVC**	45	79.9 (9.1)	41.0	94.0
**FEF**_**25-75**_**(L)**	44	3.53 (1.39)	0.75	6.34
**FEF**_**25-75**_**(%)**	44	74.1 (25.0)	16.0	124.0
**DL**_**CO**_**(mL/mmHg/min)**	34	29.7 (7.7)	11.70	44.50
**DL**_**CO**_**(%)**	34	88.0 (17.1)	52.0	136.0
**DL**_**CO**_**/VA**	34	5.0 (0.8)	3.78	6.84
**DL**_**CO**_**/VA (%)**	34	99.3 (14.7)	75.0	138.0
**VA**	34	6.0 (1.4)	2.99	8.37
**VA (%)**	34	88.8 (13.9)	52.0	117.0
**DL**_**CO**_-Hb*	34	29.8 (8.4)	11.1	43.8

SD, Standard Deviation.

*DL_CO_ corrected for Hb value.

**Table 3:** Correlations between pulmonary function parameters, total HRCT scores and HRCT pattern scores

	**FVC (n = 45)**		**FVC (%) (n = 45)**		**FEV**_**1**_**(n = 45)**		**FEV**_**1**_**(%) (n = 45)**		**FEV**_**1**_**/FVC (n = 45)**		**FEF**_**25-75**_**(n = 44)**		**FEF**_**25-75**_**(%) (n = 44)**	
	**r**	**p**	**r**	**p**	**r**	**p**	**r**	**p**	**r**	**P**	**r**	**p**	**r**	**p**
**Total HRCT score**	−0.55	0.0001	−0.49	0.001	−0.66	0.0001	−0.64	0.0001	−0.33	0.03	−0.55	0.0001	−0.53	0.0002
**MN score**	−0.34	0.02	−0.35	0.02	−0.38	0.01	−0.39	0.008	−0.04	0.79	−0.29	0.05	−0.29	0.06
**N score**	−0.60	0.0001	−0.55	0.0001	−0.63	0.0001	−0.60	0.0001	−0.20	0.19	−0.48	0.001	−0.45	0.002
**C score**	−0.35	0.02	−0.35	0.02	−0.50	0.0001	−0.56	0.0001	−0.43	0.004	−0.44	0.003	−0.47	0.001
**GGO score**	−0.38	0.009	−0.29	0.06	−0.43	0.003	−0.36	0.02	−0.17	0.26	−0.34	0.02	−0.32	0.04
**BL score**	−0.46	0.001	−0.35	0.02	−0.58	0.0001	−0.51	0.0001	−0.37	0.01	−0.49	0.001	−0.44	0.003
**SPIT score**	−0.22	0.14	−0.20	0.19	−0.24	0.11	−0.24	0.11	−0.14	0.37	−0.22	0.15	−0.19	0.22
**PB score**	−0.48	0.001	−0.40	0.006	−0.61	0.0001	−0.58	0.0001	−0.39	0.008	−0.53	0.0001	−0.50	0.001
**BVB score**	−0.13	0.38	−0.16	0.30	−0.21	0.16	−0.25	0.09	−0.11	0.46	−0.20	0.21	−0.23	0.14
	**DL**_**CO**_**(n = 34)**		**DL**_**CO**_**(%) (n = 34)**		**DL**_**CO**_**/VA (n = 34)**		**DL**_**CO**_**/VA% (n = 34)**		**VA (n = 34)**		**VA% (n = 34)**		**DL**_**CO**_-Hb* (n = 34)	
	**r**	**p**	**r**	**P**	**r**	**p**	**r**	**p**	**r**	**p**	**r**	**p**	**r**	**p**
**Total HRCT score**	−0.40	0.018	−0.33	0.06	0.10	0.59	0.13	0.45	−0.51	0.002	−0.53	0.001	−0.41	0.015
**MN score**	−0.08	0.66	−0.06	0.74	0.17	0.34	0.17	0.34	−0.18	0.30	−0.23	0.20	−0.09	0.62
**N score**	−0.41	0.016	−0.29	0.10	0.21	0.23	0.22	0.21	−0.58	0.0004	−0.54	0.001	−0.41	0.016
**C score**	−0.25	0.15	−0.21	0.24	0.08	0.64	0.10	0.56	−0.32	0.066	−0.32	0.06	−0.25	0.15
**GGO score**	−0.44	0.008	−0.38	0.03	−0.02	0.92	−0.01	0.95	−0.49	0.003	−0.46	0.006	−0.44	0.009
**BL score**	−0.46	0.006	−0.40	0.02	−0.05	0.78	−0.01	0.95	−0.50	0.002	−0.52	0.002	−0.47	0.005
**SPIT score**	−0.04	0.81	−0.08	0.66	0.02	0.92	0.02	0.92	−0.09	0.61	−0.15	0.40	−0.03	0.85
**PB score**	−0.44	0.009	−0.38	0.03	0.06	0.75	0.10	0.57	−0.54	0.001	−0.57	0.0005	−0.47	0.005
**BVB score**	0.14	0.44	0.16	0.38	0.24	0.17	0.17	0.34	−0.1	0.57	−0.1	0.58	0.12	0.51

*BL*, Bronchial lesions; *BVB*, Bronchovascular bundle; *C*, Consolidation; *GGO*, Ground glass opacity; *MN*, Micronodule; *N*, Nodule; *SPIT*, Subpleural interstitial thickening; *PB*, Parenchymal band.

*DL_CO_ corrected for Hb value.

At Chest X-ray evaluation 22 patients had radiologic stage 1, 14 patients stage 2, 4 patients stage 3 and 2 patients stage 4. Comparing the HRCT scores among the different chest-X-ray stages, the patients at radiological stage I had significantly lesser mean total HRCT score than those in stages II, III and IV, respectively (Table 4). In addition, there were significant correlations between chest-X-ray stage and pulmonary function tests (Table 5). A significant correlation between chest-X-ray stage and total HRCT score was also found (rho:0.72, p = 0.0001).

**Table 4:** Mean total HRCT scores for Chest-X-ray stages

**n**	**Chest-X-ray staging**	**Total HRCT score**
22	1	11.1
14	2	32.0
4	3	30.0
2	4	48.5

**Table 5:** Correlations between chest-X-ray stages and pulmonary function tests

	**Chest-X-ray stage**	
	**rho**	**p**
FVC (n = 42)	−0.60	0.0001
FVC (%) (n = 42)	−0.54	0.0003
FEV_1_ (n = 42)	−0.53	0.0003
FEV_1_ (%) (n = 42)	−0.54	0.0002
FEV_1_/FVC (n = 42)	0.14	0.37
FEF_25-75_ (n = 41)	−0.28	0.076
FEF_25-75_ (%) (n = 41)	−0.25	0.11
DL_CO_ (n = 31)	−0.31	0.087
DL_CO_ (%) (n = 31)	−0.24	0.20
DL_CO_/VA (n = 31)	0.23	0.21
DL_CO_/VA (%) (n = 31)	0.15	0.41
VA (n = 31)	−0.51	0.003
VA% (n = 31)	−0.41	0.021
DL_CO_-Hb (n = 31)	−0.26	0.16

Among the 44 patients whose lymph nodes were evaluated, there was only one patient with no lymph node involvement on thorax CTs. All other patients had several amounts of lymphadenopathies on their CTs. The degree of lymph node involvement was mild in 12 patients, moderate in 18 , and severe in 13.

Twenty two patients (50%) had lymph nodes enlargement in all, previously defined stations, that is the paratracheal, subcarinal, precarinal, right hilar, left hilar, paraaortic and subaortic areas. The frequency of lymph node involvement in each station was as follows : paratracheal in 43 patients (98%), subcarinal in 42 (95%), right hilar in 41 (93%), left hilar in 40 (91%), para-aortic in 38 (86%), precarinal in 31 (70%), and subaortic in 29 (66%) patients. Thus, paractracheal lymph nodes were the most frequently involved (Table 6).

**Table 6:** Involvement of lymph node stations

**Involvement of lymph node stations**	**N = 44**	**%**
Paratracheal	43	98
Subcarinal	42	95
Right hilar	41	93
Left hilar	40	91
Para-aortic	38	86
Precarinal	31	70
Subaortic	29	66
All regions	22	50
No involvement	1	2

## Discussion

In this study we have shown that MNs pattern is the most common among the wide range of abnormalities found in HRCT of patients with pulmonary sarcoidosis. This should not be surprising, because theoretically MNs can be considered the fundamental finding of parenchymal involvement in pulmonary sarcoidosis. In fact, by definition, sarcoidosis is a systemic granulomatous disease mainly involving lymphatic system, such as the formation and presence of MNs are to be expected [1,5,8,12]. Supporting our hypothesis, studies have shown that parenchymal biopsies obtained from MNs in sarcoidosis patients revealed non-caseating granulomas. It is also well known that the shape of granulomas is usually round and their dimensions may vary from micrometers to millimeters [2,8,13,14]. Also, due to systemic nature of the disease, every single organ or system might be involved to some extent in sarcoidosis, even though we could not visualize all these possible localizations of the disease by conventional imaging/screening methods, because they can be too small in some organs or systems to be visualized with these techniques [3,15]. From this point of view, we may speculate that easy and repeatable lung imaging by conventional methods might have led us to consider the lungs to be the most involved organs in sarcoidosis even though this might not be true. Furthermore, when compared to conventional methods, HRCT is more sophisticated to visualize lung parenchyma in a detailed manner; and HRCT has the capability to reveal very subtle or minimal involvements/changes in the lung parenchyma [16,17]. As a result, the demonstration of MNs might be considered as a good example for this condition in sarcoidosis patients. In many studies it was shown that the distribution of MNs was perilymphatic in HRCTs of patients with pulmonary sarcoidosis [3,5,8,14-16]. That is : MNs are mostly located in sub pleural regions, in broncho vascular bundles and sometimes in interlobular septae [3,8,16] indicating that the disease is related to lymphatic system from another point of view. This kind of distribution of MNs can also be seen in patients with lymphangitis carcinomatosa and amiloidosis [10,18]. The distribution of MNs was also perilymphatic in our study patients. We can also hypothesize that the amount of MNs might reflect, to some degree, the extent of the sarcoidosis involvement in the lungs. Compatible with this hypothesis, we have shown that there were significant correlations between spirometric values; FVC, FVC%, FEV_1_, FEV_1_%, FEF_25-75_ and FEF_25-75_% and MN scores. This finding might suggest that the more is the amount of the MNs , the less the spirometric values are. This finding indirectly suggests that the loss of pulmonary function in pulmonary sarcoidosis is related to the amount of MNs in HRCT since it is very well known that these spirometric values globally might reflect the degree of the impairment in lung function [19].

In addition, perilymphatic distribution of MNs might also contribute to their limiting effects on lung function since anatomically the hypothetical skeleton of the lungs could be regarded as involved by this kind of distribution.

Nodules were defined as rounded opacities more than 3 mm and less than 10 mm in diameter. They can form mainly in two ways :growth of a MN with time , or confluence of several MNs [5,10]. From this point of view we can propose that nodules reflect a more advanced disease stage than MNs. However, whatever the way they are formed, the nodules are supposed to have the similar appearance as MNs on HRCT, apart from their dimensions. The distribution of nodules was usually perilymphatic and similar to MNs in our patients.

Twenty one patients had some degree of consolidation on their HRCTs. Consolidation can form as an acute disease or as a result of confluence of nodules; fibrotic masses can also be considered as consolidation [5,8,10,17]. Consolidation is not infrequent but a confusing finding in differential diagnosis of the sarcoidosis since it mimics many diseases such as pneumonia and irregular malignant masses. Sometimes, consolidated areas in the lung parenchyma look like scattered stars resembling a galaxy, and for this reason such pattern is called “galaxy sign” [5,13]. This finding might also mimic metastatic lung cancer [17,20]. Furthermore, pulmonary sarcoidosis presenting predominantly with consolidation is also called alveolar sarcoidosis [3,5,13,16,17]. Consolidation is a lesion occupying space in the lungs, thus the presence of consolidation might be expected to inversely associate with the results of lung function tests. Contributing to this hypothesis significant and negative correlations were found in the present study between consolidation scores and spirometric values : FVC, FVC%, FEV_1_, FEV_1_%, FEF_25-75_, FEF_25-75_%.

GGO can form mainly as a result of certain diseases or conditions characterized by accumulation of fluid, blood, inflammatory or malignant cells in alveoli, such as alveolar hemorrhage, alveolar proteinosis, viral pneumonia, interstitial lung diseases etc. [10]. However, in sarcoidosis the confluence of granulomas was reckoned to be responsible for GGO appearance on HRCT rather than a true alveolitis [5,13,16]. It sometimes surrounds consolidation which is called as "halo sign" and when its surrounded by consolidation it is called as "reversed halo sign". These findings could also be seen in a variety of disorders such as fungal infections and organizing pneumonia [21-23]. GGO might also be considered as a light or less dense form of consolidation. GGO was found in most our patients. Since alveoli are the main site involved in GGO appearance and they are the main site of gas exchange, we can hypothesize that the presence of GGO might be inversely related to gas exchange parameters. Compatible with our hypothesis, a significant and negative correlation between GGO scores, DL_CO_ and DL_CO_% values were found in the present study. In addition, significant and negative correlations were found between GGO scores and spirometric values.

When combining all our findings, we might say that the correlations between MN and N scores and reduction in lung volumes are striking. This result suggests that an increase in the number of micronodules and/or nodules may determine a reduction in lung volumes.

Total HRCT score can be supposed to reflect the degree of pulmonary parenchymal involvement more than any single parenchymal lesion in pulmonary sarcoidosis patients since it includes almost all the parenchymal lesions seen on HRCT. Compatible with this hypothesis significant and negative correlations were found between total HRCT scores and PFT parameters indicating reduced lung volumes. This finding suggests that rather than any single lesion such as MN or N, a combination of all lesions seen in HRCT can be strongly related to the reductions in lung volumes in sarcoidosis patients.

In this study we have also shown that staging of the disease with respect to conventional chest-x-ray did not reflect the status of actual parenchymal involvement in patients with sarcoidosis. In fact, although there were 22 patients with sarcoidosis at radiologic stage 1 according to chest-X-rays, nevertheless only few patients had almost clear parenchymal HRCT images. The low sensitivity of chest-x-ray in detecting subtle parenchymal changes in lung parenchyma likely is the main reason for this finding. Before the introduction of HRCT for the imaging of lungs, chest-X-ray had been used for an aid to the evaluation, staging and monitoring of the disease along with spirometric and diffusion tests [3,7,16]. However the interpretation of chest-X-ray might be limited by many factors such as weight, position and the degree of inspiration of the patient, the dose of the beam etc. In addition, the superimposition of many intrathoracic structures is another limiting factor. From this point of view, our finding should not be surprising but an expected one. Several new studies indicate the low value of chest-x-ray in clinical decision making in patients with parenchymal lung diseases [3,7,16,17,24]. On the other hand, clinical usefulness of chest-X-ray should not be discarded in pulmonary sarcoidosis since patients with radiological stage I disease had significantly less mean total HRCT score than patients with other chest x-ray stages. Furthermore, we have found significant correlations between radiologic stages of the disease and total HRCT scores. These findings might be interpreted as following : even though chest-X-ray does not necessarily reflect the degree of actual involvement in pulmonary parenchyma, it has a considerable value for the estimation of the existence of pulmonary involvement. Supporting the aforementioned interpretation, we have also found significant correlations between chest-X-ray scores and pulmonary function test parameters. As a whole, our results suggest that chest-X-ray along with pulmonary function tests might be used to determine the functional status of pulmonary sarcoidosis patients. However these findings should not induce to consider that chest-X-ray is comparable to computed tomography or that it can be used to monitor the disease.

Compatible with the current literature [3,5,8,17], we have also shown that lymph nodes in paratracheal area were the most involved lymph nodes, preceding subcarinal, and other lymph node stations. Except one patient all patients had lymph nodes in both hilar regions. There was a strong correlation between chest-X-ray hilar score values and CT LAP score values suggesting that chest-X-ray might be used to estimate the amount of lymphadenopathies without using computed tomography.

Although the retrospective nature of our study might be possible limitation, we believe it is informative since we tried to document the findings obtainable in patients with pulmonary sarcoidosis by means of pulmonary function tests and CT scans and the possible relationships between them, that we also interpreted in the light of the current literature.

## Conclusions

In conclusion, we have shown that pulmonary sarcoidosis patients may have various pulmonary parenchymal changes, also seen in other interstitial lung diseases. Even though it is an expected finding and its clinical significance is debatable, we have also shown that thorax HRCT was significantly superior to chest-X-ray in detecting pulmonary parenchymal abnormalities. Nevertheless, our results also suggest that chest-X-ray along with pulmonary function tests might be used to determine the functional status of patients with pulmonary sarcoidosis. We have also shown that the degree of pulmonary involvement can be closely related to the loss of pulmonary function as measured by PFTs. The combination of multiple parenchymal lesions, represented in our study by total HRCT score, seems more responsible for the loss of pulmonary function than every individual pathological alteration.

## Competing interests

The authors declare that they have no competing interests.
